# Comprehensive GC-MS Characterization and Histochemical Assessment of Various Parts of Three *Colchicum* Species from Bulgarian Flora

**DOI:** 10.3390/plants14020270

**Published:** 2025-01-18

**Authors:** Ivayla Dincheva, Ilian Badjakov, Vasil Georgiev, Ivanka Semerdjieva, Radka Vrancheva, Ivan Ivanov, Atanas Pavlov

**Affiliations:** 1 Department of Agrobiotechnologies, Agrobioinstitute, Agricultural Academy, 8 Dr. Tsankov Blvd., 1164 Sofia, Bulgaria; ivadincheva@abi.bg (I.D.); ibadjakov@abi.bg (I.B.); 2 Laboratory of Cell Biosystems, Institute of Microbiology, Bulgarian Academy of Sciences, 139 Ruski Blvd., 4000 Plovdiv, Bulgaria; vasgeorgiev@microbio.bas.bg; 3 Department of Botany and Agrometeorology, Faculty of Agronomy, Agricultural University, 4000 Plovdiv, Bulgaria; v_semerdjieva@au-plovdiv.bg; 4 Department of Plant and Fungal Diversity, Division of Flora and Vegetation, Institute of Biodiversity and Ecosystem Research, Bulgarian Academy of Sciences, 1113 Sofia, Bulgaria; 5 Department of Analytical Chemistry and Physical Chemistry, University of Food Technologies, 26 Maritza Blvd., 4002 Plovdiv, Bulgaria; r_vrancheva@uft-plovdiv.bg; 6 Department of Organic Chemistry and Inorganic Chemistry, University of Food Technologies, 26 Maritza Blvd., 4002 Plovdiv, Bulgaria; i_ivanov@uft-plovdiv.bg

**Keywords:** *Colchicum autumnale* L., *Colchicum bivonae* Guss., *Colchicum diampolis* Delip. et Česchm., GC-MS, histochemical analysis, medicinal plants, phytochemical profiling

## Abstract

This study presents a comprehensive phyto- and histochemical analysis of three *Colchicum* species: *Colchicum autumnale* L., the Balkan endemic *Colchicum bivonae* Guss., and the Bulgarian endemic *Colchicum diampolis* Delip. et Česchm. Using gas chromatography-mass spectrometry (GC-MS), 66 metabolites were identified, encompassing free amino, organic, phenolic, and fatty acids, sugars, and alkaloids, which were distributed among various plant parts. Organ-specific metabolic patterns revealed that corms and seeds are particularly rich in alkaloids, supporting their roles in chemical defense and survival during dormancy. Conversely, flowers, leaves, and capsules were enriched with energy-related and phenolic compounds, playing critical roles in reproduction and stress tolerance. Histochemical investigations localized alkaloids predominantly in the endosperm of seeds, parenchyma of corms, and vascular bundles of flowers. Notably, the endemic *C. bivonae* and *C. diampolis* displayed unique chemical profiles. Moderate acetylcholinesterase inhibitory activity (AChE) was observed across various plant organs. Statistical analyses demonstrated significant interspecies and organ-specific chemical differentiation, with certain metabolites serving as key markers. These findings enhance our understanding of the chemical composition, organ specialization, and potential as a source of new biomolecules in these *Colchicum* species. They underscore the ecological and pharmacological importance of endemic taxa and provide a framework for future research into their sustainable utilization and potential bioactivities.

## 1. Introduction

Medicinal plants have captivated human interest for thousands of years, serving as a vital component of healthcare from ancient times to the present. Their enduring significance is evident, with over 50,000 plant species currently used worldwide for their healing properties. These plants form the foundation of both traditional remedies and pharmaceutical innovations. According to the World Health Organization (WHO), over 40% of approved pharmaceutical products are derived from plants, with 25% originating from higher plants, highlighting their crucial contribution to modern medicine [1]. Regions like South-Eastern Europe are rich in medicinal plant species, making them an essential resource for traditional and modern healthcare. Bulgaria is renowned for its diverse medicinal flora and ethnobotanical heritage, which hold considerable cultural, social, and economic value.

Among ethnobotanical flora, species of the *Colchicum* (commonly referred to as autumn crocus, meadow saffron, or naked lady) are notable for their medicinal applications. These plants owe their therapeutic potential primarily to tropolone alkaloids, with colchicine being the most prominent bioactive compound. Known for its remarkable anti-inflammatory and anti-mitotic effects, colchicine has been widely utilized in both traditional medicine and modern pharmacology, particularly in the treatment of disorders such as amyloidosis [2], familial Mediterranean fever [3,4], cirrhosis [5], Behcet’s disease [6], psoriasis and dermatological ailments [7], Hodgkin’s lymphoma, acute myeloid leukemia, cancer chemotherapy, pain management, gout prevention, and treatment [8,9]. Recent findings provide a basis for colchicine’s promising role in mitigating critical complications of COVID-19, particularly its potential to modulate the hyper-inflammatory responses characteristic of severe cases [10]. In addition, pharmacological research highlights the diverse biological activities of *Colchicum* species, including antioxidant, antibacterial, acetylcholinesterase-inhibitory, and anti-inflammatory effects [11,12]. The genus *Colchicum* L. (Colchicaceae) comprises approximately 90 geophytic species [13]. Its distribution extends from North America, Asia, North Africa, Europe, and Eurasia [14]. Generally, in Bulgarian flora, there are 10 species documented [15], with five protected under the Biodiversity Act. These include both spring-flowering (*C. diampolis*, *C. doerfleri* Halascy, *C. davidovii* Stef.) and autumn-flowering species (*C. bivonae*, *C. autumnale*, *C. turcicum* Janka, *C. haynaldii* Heuff.). The fruits of *Colchicum* species are a three-part capsule, facilitating seed disperses [15].

The most widespread is *C. autumnale*, which produces 3–7 pink to purple flowers per plant, with linear-lanceolate to oblong-elliptic leaves [15].

In contrast, *C. bivonae*, an endangered [EN B1ab(ii)] and protected species, Balkan endemic, inhabits fragmented xerothermic habitats and is included in the European ecological network NATURA 2000 [16]. Its flowers range from light to dark pink-purple with checkered patterns, and its corms are large and egg-shaped, covered with dark brown tunics. These plants exhibit a unique life cycle, alternating between flowering and fruit development in late summer, followed by dormancy, and leaf and fruit formation in the spring [17]. *C. autumnale* and *C. bivonae* begin flowering in late summer (August–September), after which they enter a period of dormancy. In the following spring, the plants produce their leaves and fruits. The corm, a modified underground stem, plays a vital role during dormancy by storing essential nutrients and water. This storage ensures the plant’s survival and supports its growth when active development resumes. Additionally, the corm enables vegetative propagation, allowing the species to reproduce asexually [16].

*Colchicum diampolis* is a perennial, critically endangered Bulgarian endemic species [CR B1ab(ii)+2ab(iii)] [18]. It is also protected under the Biodiversity Act. The species has a small, ovate corm covered with dark brown tunics [18]. Its flowers range from pink to white, with the flowering period from early February to March. *C. diampolis* inhabits highly fragmented areas, primarily in wet meadows and riparian mixed forests [18].

According to the available literature, no comprehensive studies have been undertaken to analyze the metabolites across different plant parts (capsules, flowers, leaves, corms, and seeds) of *C. autumnale* and two endemic species, *C. diampolis*, and *C. bivonae*. This investigation aims to fill this gap by utilizing untargeted metabolomics techniques, specifically Gas Chromatography coupled with Mass Spectrometry (GC-MS). Using this approach, the study aims to outline a detailed profile of the metabolites in these plant parts, highlighting their chemical diversity and potential functional significance. The research investigates the metabolic diversity in various parts of three *Colchicum* species, aiming to identify key metabolites that could act as chemotaxonomic markers associated with specific organs and species. Additionally, in vitro enzyme inhibitory assays were performed to assess the acetylcholinesterase inhibitory activity regarding the pharmaceutical significance of these plants. The tests provide a deeper understanding of the therapeutic potential of the metabolites, emphasizing their prospective applications in drug discovery and development.

## 2. Results

### 2.1. Histochemical Analysis for the Localization of Alkaloids

Histochemical analysis using Dragendorff’s reagent confirmed the presence of alkaloids in the flowers, corms, and seeds of species analyzed. The reagent produced distinct staining patterns, ranging from orange to yellow, highlighting alkaloid-rich tissues in these plant parts. Overall, all studied *Colchicum* species corms displayed a pale, reddish-brown coloration. The corm structure consisted of single-layered epidermal cells with square-shaped cells and layers of parenchymal cells containing starch grains, with vascular bundles scattered throughout these parenchymal layers.

The seeds of these species are subglobose to globose in shape and brown colors with proliferation in the basal part, most pronounced in *C. autumnale*. The spermoderms of the three target species are rugose, with irregular and flattened cells with brown pigments. The endosperm consists of wide and long rectangular parenchyma cells with thickened cell walls and numerous pits. The parenchyma cells contain a large number of aleurone grains and lipid droplets.

**Figure 1:**
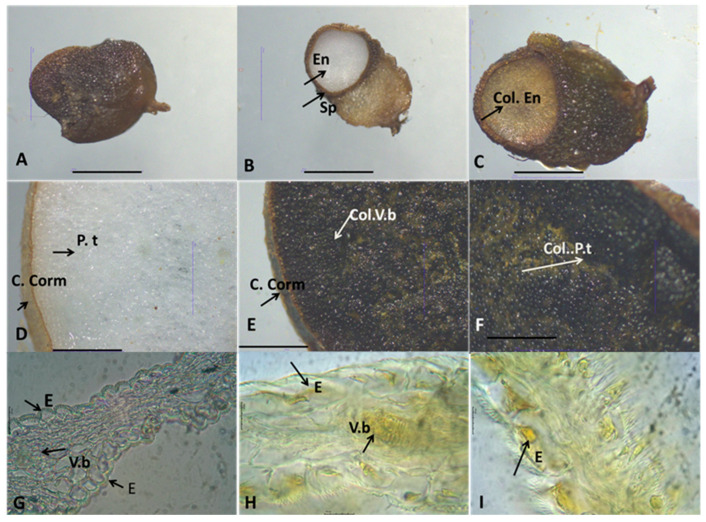
Morphological features of seeds, corms, and flowers (perianth leaves) of *Colchicum autumnale* viewed with Stereo Microscope Motic DM 143 (**A**–**F**) and light microscope Motic DMA (**G**–**I**; (**G**) ×10; (**H**,**I**); ×40); (**A**)—general view of seeds; (**B**)—non treated seeds; (**C**)—treated seeds; (**D**)—non treated corm, Control; (**E**,**F**)—treated corm; (**G**)—perianth leaves, Control; (**H**,**I**)—treatment perianth leaves; En—endosperm; Sp—spermoderm; Col. En—colored endosperm; C. Corm—cortex of corm; P.t—parenchyma tissue; Col. V.b—colored Vascular bundles; Col. P.t—colored parenchyma tissue E—epidermis; V.b—Vascular bundles; Scale bar = 100 μm.

**Figure 2:**
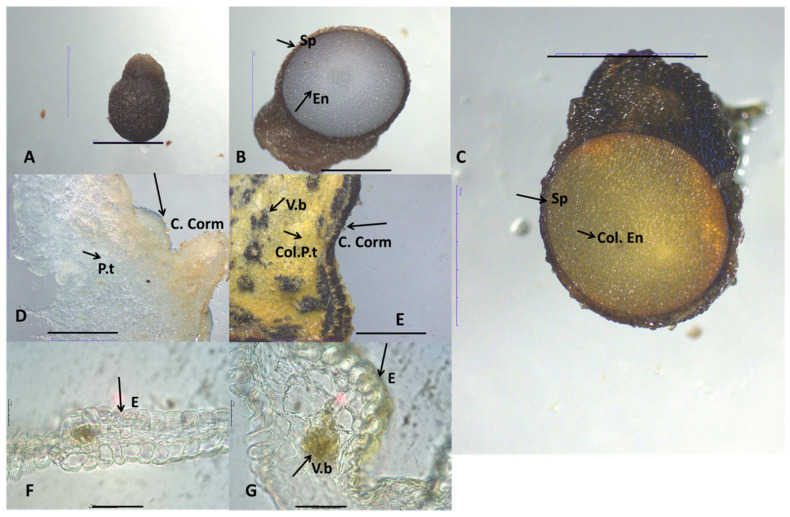
Morphological features of seeds, corm, and flowers (perianth leaves) of *Colchicum bivonae* viewed with Stereo Microscope Motic DM 143 (**A**–**F**) and light microscope Motic DMA ((**F**) ×10, (**G**) ×40); (**A**)—general view of seeds; (**B**)—non-treated seeds, Control; (**C**)—treated seeds; (**D**)—non-treated corm, Control; (**E**)—treated corm; (**F**)—perianth leaves, Control; (**G**)—treated perianth leaves; En—endosperm; Sp—spermoderm; Col. En—colored endosperm; C. Corm—cortex of corm; P.t—parenchyma tissue; Col. P.t—colored parenchyma tissue E—epidermis; V.b—Vascular bundles; Scale bar = 100 μm.

**Figure 3:**
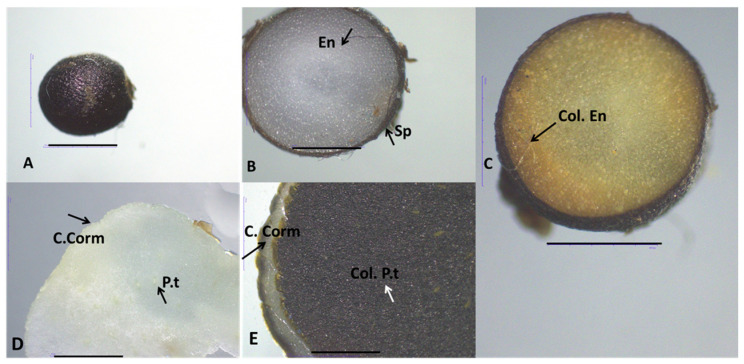
Morphological features of seeds and corm of *Colchicum diampolis* viewed with Stereo Microscope Motic DM 143 (**A**–**E**); (**A**)—general view of seeds; (**B**)—non-treated seeds, Control; (**C**)—treated seeds; (**D**)—non-treated corm, Control; (**E**)—treated corm; En—endosperm; Sp—spermoderm; Col. En—colored endosperm; C. Corm—cortex of corm; P.t—parenchyma tissue; Col. P.t—colored parenchyma tissue; Scale bar = 100 μm.

Unstained samples of these plant organs were used as controls (Figure 1, Figure 2 and Figure 3). Generally, the alkaloids in studied *Colchicum* species were detected in the endosperm (Col. En) of seeds, parenchyma tissues (P.t), and vascular bundles (V.b) of corms (Figure 1C,E,F, Figure 2 and Figure 3C,E).

Microscopic examination of *C. autumnale* L. flowers showed the presence of alkaloids in the epidermis cells and vascular bundles (V.b) (Figure 1H,I). Analysis of the *C. bivonae*. flowers revealed low alkaloid levels, primarily concentrated in the vascular bundles (Figure 2G).

### 2.2. Comprehensive GC-MS Characterization and Multivariate Statistical Discrimination of Various Parts of Three Colchicum Species

A total of 66 metabolites, encompassing various chemical classes such as free amino acids, organic acids, phenolic acids, fatty acids, sugars, and alkaloids, were identified across different organs of *Colchicum* species, including corms, seeds, flowers, leaves, and fruit capsules (Table S1), using the GC-MS approach. Of these, 13 compounds—namely isoleucine, leucine, valine, malic acid, fructose, glucose, sucrose, glucitol, benzoic acid, palmitic acid, linoleic acid, and colchicine—were confirmed using authentic standards. The remaining metabolites were tentatively identified through the NIST’08 mass spectral library, the Golm Metabolome Database, and a custom in-house library. Quantification of the compounds was performed through the internal standard method.

The analyses revealed distinct metabolite profiles for each plant part. This organ-specific metabolic specialization underscores the varied functional roles of the corms, seeds, flowers, leaves, and fruit capsules in the plant’s growth, reproduction, and survival strategies.

This research explored the metabolic diversity across different organs of three *Colchicum* species, aiming to identify key metabolites that could serve as chemotaxonomic markers linked to specific organs and species.

#### 2.2.1. Multivariate Statistical Discrimination of Various Parts of *Colchicum autumnale* Organs

**Figure 4:**
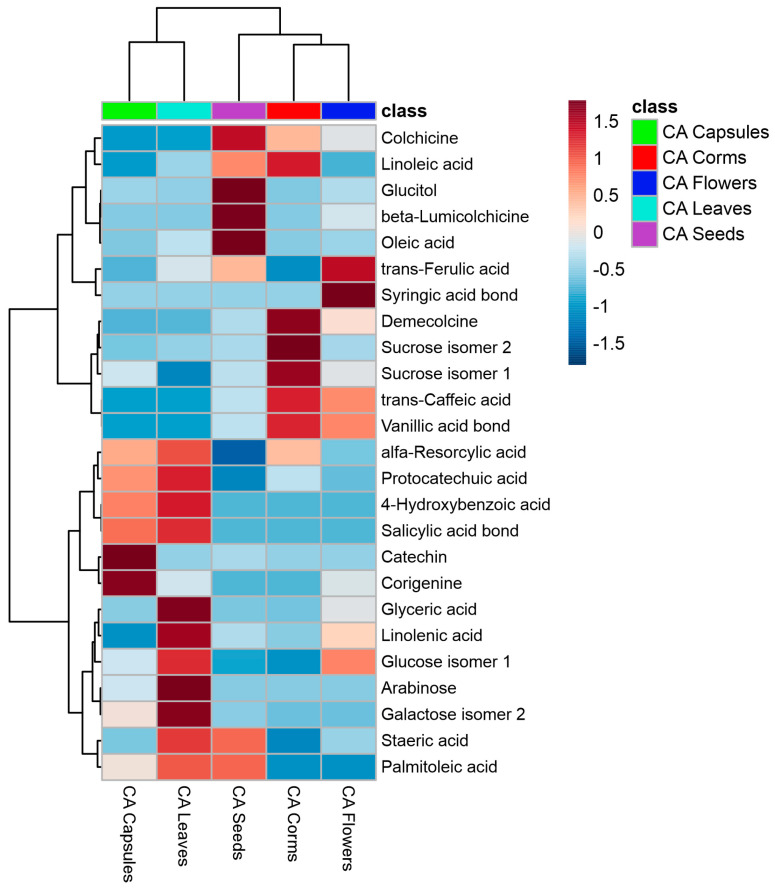
Heatmap of 25 most significant (ANOVA) GC/MS identified metabolites in different *Colchicum autumnale* organs: corms (red); capsules (green); flowers (dark blue); leaves (light blue); and seeds (pink).

To outline the most significant differences in metabolite profiles of the investigated *C. autumnale* organs, we used a hierarchical clustered heatmap analysis of the 25 most significant metabolites according to a one-way ANOVA post hoc test with a cutoff value *p* = 0.05. The results are presented in Figure 4.

The clustering was performed by using the Ward method with Euclidean distance. The samples are in columns and the metabolites are in rows, mapped with the highest (red) and lowest (blue) levels.

**Figure 5:**
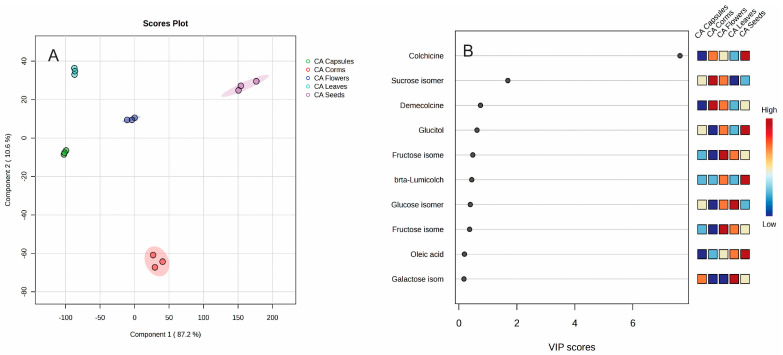
PLS-DA score plot (**A**) and variable importance analysis (VIP) (**B**) of top 10 GC/MS identified metabolites in different *Colchicum autumnale* organs.

Different organs can be clearly distinguished by their metabolite content. The capsules are abundant in catechin and corigenine; the leaves contain glyceric acid, linolenic acid, arabinose, and galactose; the seeds are rich in glucitol, beta-lumicolchicine, and oleic acid; the corms are notable for demecolcine and sucrose; and the flowers are characterized by syringic acid. To find the most important metabolites for distinguishing the different plant organs, a Partial Least Squares Discriminant Analysis (PLS-DA) analysis was performed (Figure 5).

The partial least squares discriminant analysis (PLS-DA) was successfully applied to discriminate different plant organs into five groups. Analyses of variable importance showed that colchicine (VIP score = 7.6308), sucrose (VIP score = 1.6898), and demecolcine (VIP score = 0.7448) are the most significant variables.

#### 2.2.2. Multivariate Statistical Discrimination of Various Parts of *Colchicum bivonae* Organs

**Figure 6:**
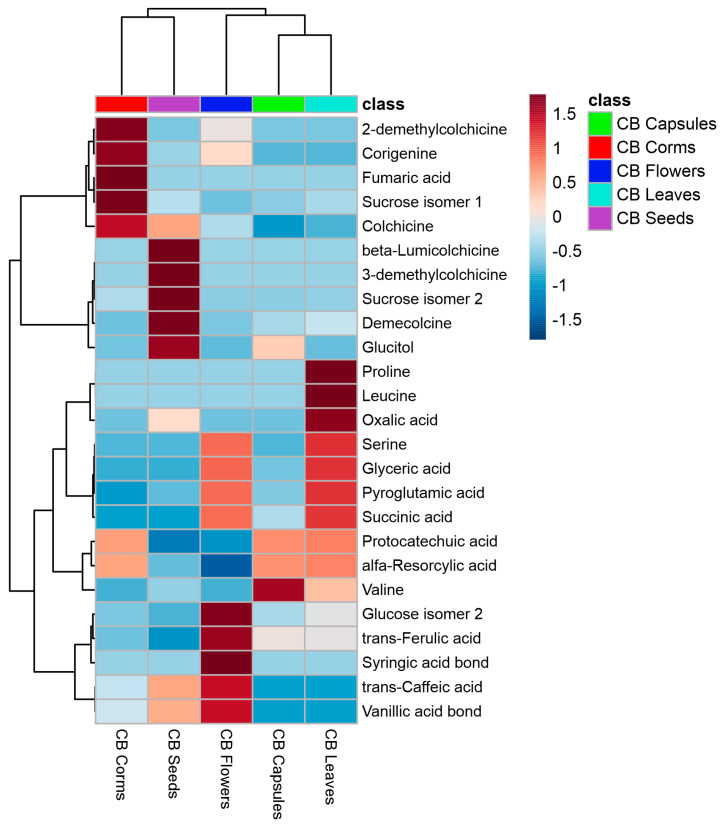
Heatmap of 25 most significant (ANOVA) GC/MS identified metabolites in different *Colchicum bivonae* organs: corms (red); capsules (green); flowers (dark blue); leaves (light blue); and seeds (pink).

A Heatmap displaying the 25 most significant metabolites (one-way ANOVA, *p* = 0.05) identified in the organs of *C. bivonae* is shown in Figure 6.

**Figure 7:**
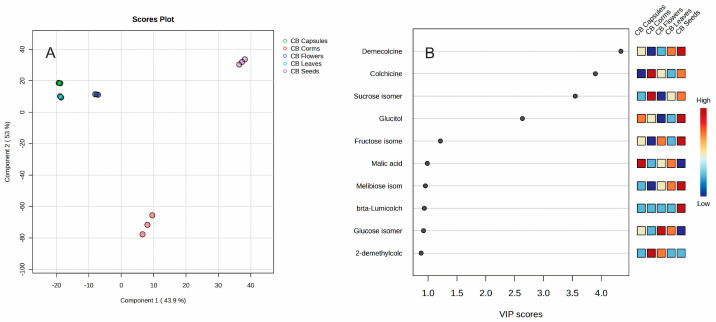
PLS-DA score plot (**A**) and variable importance analysis (VIP) (**B**) of top 10 GC/MS identified metabolites in different *Colchicum bivonae* organs.

Corms and seeds formed one cluster, while capsules, leaves, and flowers grouped into a second cluster. The corms exhibited elevated concentrations of salicylic alcohol, fumaric acid, sucrose, 2-demethylcolchicine, and corigenine. Seeds were enriched with β-lumicolchicine, 3-demethylcolchicine, demecolcine, and sucrose. Leaves showed elevated levels of leucine and oxalic acid, capsules were abundant in valine, and flowers contained higher concentrations of syringic acid, trans-ferulic acid, trans-caffeic acid, and vanillic acid. Partial Least Squares Discriminant Analysis (PLS-DA) identified the most significant metabolites differentiating the various *C. bivonae* organs. (Figure 7).

Different *C. bivonae* organs were organized into five groups. Analyses of variable importance showed that demecolcine (VIP score = 4.3413), colchicine (VIP score = 3.8997), sucrose (VIP score = 3.5499), and glucitol (VIP score = 2.6353) were the most significant variables.

#### 2.2.3. GC-MS Profiling of *Colchicum diampolis* Organs

**Figure 8:**
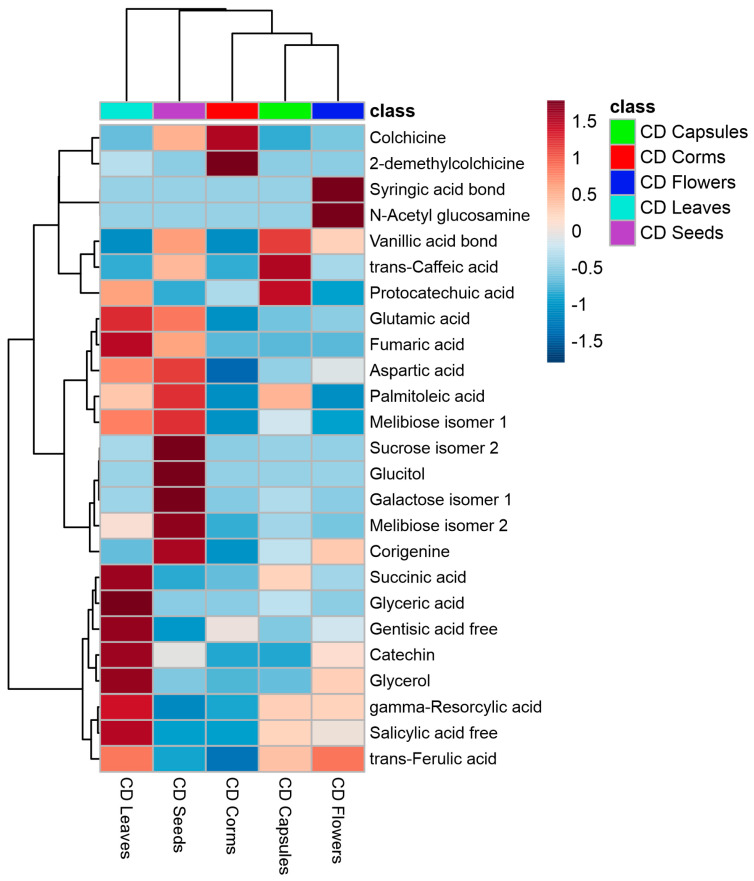
Heatmap of 25 most significant (ANOVA) GC/MS identified metabolites in different *Colchicum diampolis* organs: corms (red); capsules (green); flowers (dark blue); leaves (light blue); and seeds (pink).

Twenty-five of the most significant metabolites (one-way ANOVA, *p* = 0.05), were established in different *C. diampolis* organs were organized using heatmap visualization (Figure 8).

**Figure 9:**
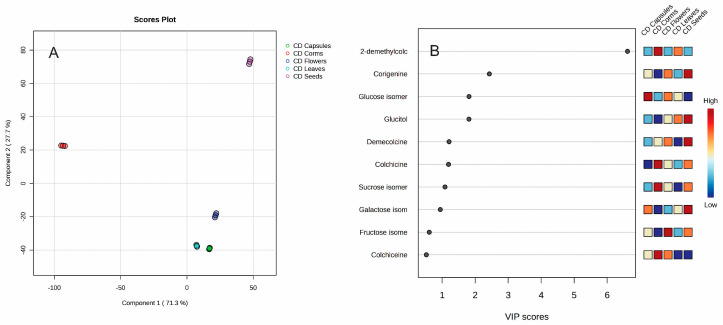
PLS-DA score plot (**A**) and variable importance analysis (VIP) (**B**) of top 10 GC/MS identified metabolites in different *Colchicum diampolis* organs.

The clustering analysis could not distinctly separate the organs into clear clusters but showed that capsules and flowers share closer similarities compared to other organs. Capsules were characterized by high levels of trans-caffeic and protocatechuic acids, while flowers were enriched in syringic acid and N-acetyl glucosamine. In contrast, corms contained abundant colchicine and 2-demethylcolchicine; seeds were rich in sucrose, melibiose, galactose, glucitol, and corigenine; and leaves were characterized by succinic acid, glyceric acid, gentisic acid, salicylic acid, catechin, and glycerol. PLS-DA identified the most significant variables distinguishing these organs (Figure 9).

The VIP analyses showed that 2-demethylcolchicine (VIP score = 6.617), corigenine (VIP score = 2.4299), glucose (Vip score = 1.8137), and Glucitol (VIP score = 1.8111) were the most significant variables.

#### 2.2.4. Phytochemical Discrimination of Three *Colchicum* Species

**Figure 10:**
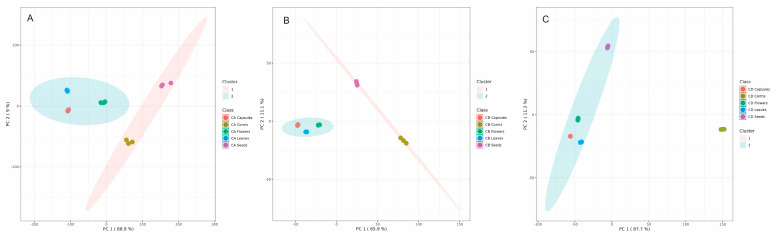
K-mean clustering (2 clusters) of different organs: corms (red); capsules (green); flowers (dark blue); leaves (light blue); and seeds (pink) of *Colchicum autumnale* (**A**), *Colchicum bivonae* (**B**), and *Colchicum diampolis* (**C**) plants.

To find the most suitable plant organs for performing phytochemical discrimination of *C. autumnale*, *C. bivonae*, and *C. diampolis.* plants, we used unsupervised machine learning (K-means clustering) to group different plant organs into two clusters based on their chemical composition (Figure 10).

**Figure 11:**
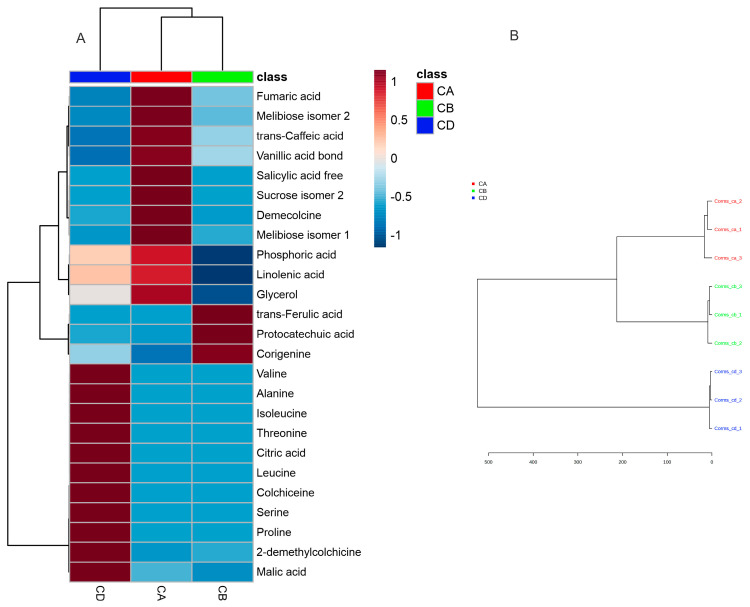
Heatmap of 25 most significant (ANOVA, *p* = 0.05) metabolites (**A**) and hierarchical clustering dendrogram of all metabolites (**B**) identified by GC/MS in corms of *Colchicum autumnale* (CA), *Colchicum bivonae* (CB), and *Colchicum diampolis* (CD). The clustering was performed by using the Ward method with Euclidean distance.

The data in Figure 10 showed that corms and seeds are organized in one cluster and leaves, capsules, and flowers in the second cluster in *C. autumnale* and *C. bivonae* plants. In *C. diampolis*., only corms are separated from the second cluster, containing seeds, leaves, capsules, and flowers. This analysis shows that the corms of the three plants can be used as an effective organ for phytochemical profiling and discrimination of the investigated species. That is why our next experiments were focused on corms (Figure 11).

**Figure 12:**
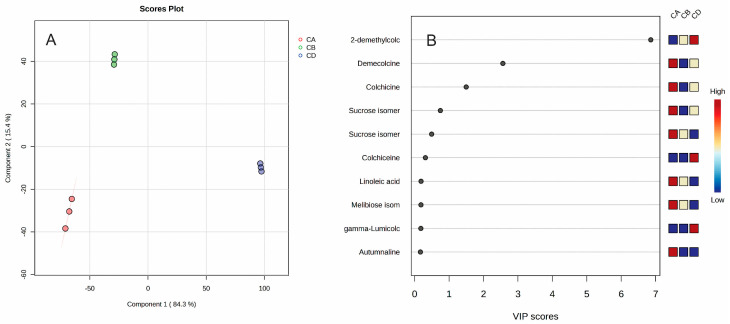
PLS-DA score plot (**A**) and variable importance analysis (VIP) (**B**) of top 10 GC/MS identified metabolites in corms of *Colchicum autumnale* (CA), *Colchicum bivonae* (CB), and *Colchicum diampolis* (CD).

The hierarchical clustering dendrogram (Figure 12) discriminates the investigated plants by the content of their metabolites. The grouping showed that *C. autumnale* (CA) and *C. bivonae* (CB) are more closely related and distinguished by *C. diampolis*. However, we used a PLS-DA analysis to find the most important metabolites in the corms, which may be used to discriminate the investigated plants.

The partial least squares discriminant analysis (PLS-DA) was able to discriminate different plants and organize them into three groups. The first component explains 84.3% and the second component explains 15.4% of the variance. The variable importance analyses showed that 2-demethylcolchicine (VIP score = 6.861), demecolcine (VIP score = 2.5637), and colchicine (VIP score = 1.4983) were the most significant variables in discriminating the plant corms. In addition, sucrose isomer 1 (VIP score = 0.74863) and sucrose isomer 2 (VIP score = 0.49483) can also be used as markers.

### 2.3. Acetylcholinesterase Inhibitory Activities of Alkaloid Extracts from Colchicum autumnale, Colchicum bivonae, and Colchicum diampolis Corms

Acetylcholinesterase (AChE) is an enzyme that rapidly hydrolyzes a neurotransmitter acetylcholine [19]. This enzyme was detected in the human brain among neurofibrillary tangles and neurotic plaques. It has been reported that low levels of acetylcholine in the human body cause different neurological disorders such as Alzheimer’s disease (AD), senile dementia, Parkinson’s disease, and others [19,20]. However, many synthetic acetylcholinesterase (AChE) inhibitors, such as tacrine and donepezil, are associated with side effects in humans and are only effective in the early stages of diseases like Alzheimer’s. As a result, research has increasingly shifted toward identifying new, effective AChE inhibitors of natural origin [21].

This study evaluated the potential of alkaloid fractions isolated from the corms of *C. autumnale, C. bivonae,* and *C. diampolis* to inhibit acetylcholinesterase enzyme (Table 1).

**Figure 13:**
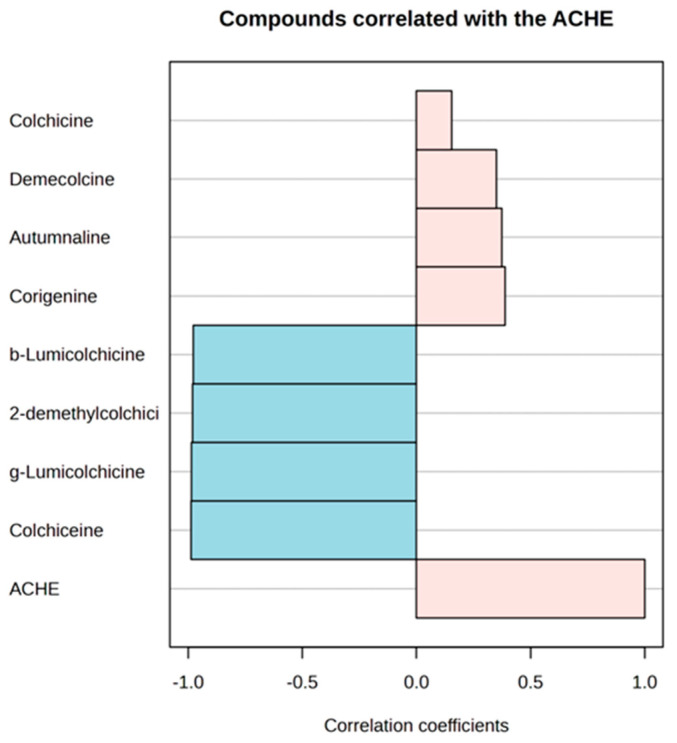
Correlation analysis showing compounds correlated with acetylcholinesterase inhibitory activities of alkaloid extracts from *Colchicum autumnale*, *Colchicum bivonae,* and *Colchicum diampolis* corms.

Among the three species tested, *C. bivonae* corms demonstrated the highest inhibition at 16.44%, indicating the strongest potential for inhibiting acetylcholinesterase. *Colchicum autumnale* corms exhibited a moderate inhibition of 14.82%, while *C. diampolis* corms showed the weakest inhibition at 11.82%. Notably, the colchicine standard showed no inhibition (0%), suggesting that pure colchicine does not affect acetylcholinesterase activity under the experimental conditions. Correlation analyses were conducted to identify which alkaloids might potentially inhibit the acetylcholinesterase enzyme (Figure 13 and Table 1).

The data show low positive correlations of acetylcholinesterase inhibitory activities with concentrations of corigenine (r = 0.38883), autumnaline (r = 0.37473), demecolcine (r = 0.35006), and colchicine (r = 0.15464), but they were statistically insignificant (*p* = 0.30102; *p* = 0.32039; *p* = 0.35573; and *p* = 0.69118).

## 3. Discussion

### 3.1. Histochemical Analysis

As part of our research on the alkaloid content in three *Colchicum* species native to the Bulgarian flora, we present, for the first time, histochemical analyses of their plant parts (corm, flowers, and seeds). As it is well-known, secondary metabolites in medicinal plants accumulate in various plant tissues [22]. Consequently, tissue- and cell-specific phytochemical profiling can provide valuable insights into the relationship between plant tissues and their chemical constituents. Furthermore, histochemical studies provide detailed tissue- and cell-specific chemical profiling which were essential for evaluating the quality of medicinal herbs and advancing research in phytochemistry and phytotherapy [23]. As is well-known, the synthesis of colchicine begins with phenethyl-isoquinoline alkaloids [24], which can be found in various parts of the plant. Similar findings were reported for Amaryllidaceae alkaloids, where the alkaloid content in *Hippeastrum papilio* (Ravena) van Scheepen was localized in vascular bundles, vacuoles, and intracellular spaces [25]. In other plant tissues and structures of *H. papilio*, the distribution of alkaloids varied depending on the specific plant organ [25].

However, it is important to note that in a given species, only one or two specific organs are primarily responsible for alkaloid production [24]. This study revealed the presence of alkaloids in the seeds and corms of *C. autumnale*, *C. diampolis*, and *C. bivonae*. In all three *Colchicum* species examined, the endosperm of the seeds and the parenchyma of the corms were identified as structures rich in alkaloids. The vascular bundles of flowers were structures where localization alkaloids were located. Enzymes involved in alkaloid biosynthesis are distributed across diverse subcellular compartments (the cytosol, vacuole, tonoplast membrane, and endoplasmic reticulum) [26]. Furthermore, they were found in plastids (chloroplast stroma, thylakoid membranes), phloem, and parenchyma tissues [26]. Numerous studies have identified parenchyma cells and vascular bundles as key structures with high concentrations of alkaloids [25,27].

### 3.2. Phytochemical Composition and Multivariate Statistical Discrimination of Various Parts of Three Colchicum Species

The metabolite distributions in different plant organs provide insight into their distinct physiological roles, showcasing the functional specialization inherent to plant biology. Considering the absence of similar comprehensive phytochemical screenings in the literature on the different plant parts of the species investigated, we aim to fill this gap by providing detailed insights into their metabolite profiles, which could contribute significantly to understanding their chemical composition and potential applications.

Corms contained significant amounts of alkaloids, phenolic acids, and organic acids. Among the species analyzed, colchicine was most concentrated in the corms of *C. autumnale* (1.83 mg/g dried weight, DW), followed by *C. bivonae* (1.24 mg/g DW) and *C. diampolis* (0.98 mg/g DW). These values align with previously reported data for *C. micranthum* (1.83 mg/g DW) and *C. chalchedonicum* (0.41 mg/g DW) [28], as well as *C. speciosum* (2.14 mg/g DW), *C. robustum* (0.49 mg/g DW), and *C. kotschyi* (0.77 mg/g DW) [29]. Additionally, studies by Rocchetti, et al. [30] documented an average total alkaloid content of 1.65 mg/g DW in *C. szovitsii* corms, meanwhile, Senizza, et al. [31] reported a higher average of 2.89 mg/g DW in *C. triphyllum* corms, emphasizing the notable abundance of tropolone alkaloids in *Colchicum* species. In our study, the total alkaloid content in the corms of the three species was measured as follows: *C. autumnale* (2.44 mg/g DW), *C. diampolis* (1.43 mg/g DW), and *C. bivonae* (2.72 mg/g DW). The less toxic colchicine analog demecolcine was detected at 0.57 mg/g DW in *C. autumnale* corms, while 2-demethylcolchicine was the most abundant in *C. diampolis* corms (1.31 mg/g DW). The reduced toxicity of these compounds compared to colchicine underscores their pharmaceutical potential. In contrast, 3-demethylcolchicine was present only in trace amounts, consistent with its classification as a minor alkaloid. The total phenolic acid content in the corms was recorded as 3.33 mg/g DW for *C. autumnale*, 2.10 mg/g DW for *C. diampolis*, and 3.06 mg/g DW for *C. bivonae*, respectively. These findings align with those of Rocchetti, et al. [30], who reported levels ranging from 2.55 to 5.19 mg/g DW in *C. szovitsii* corms, depending on the extraction method. Organic acids, including malic and fumaric acid, were also identified in notable amounts.

The seeds of three *Colchicum* species exhibited a distinctive metabolite profile, characterized by significant levels of sugar alcohols, alkaloids, and fatty acids. The sugar alcohol, glucitol, which plays a crucial role in maintaining cellular hydration and preserving seed viability under stress, was abundant across all species. The highest glucitol concentration was observed in *C. bivonae* seeds (27.21 mg/g DW), followed by *C. diampolis* (19.84 mg/g DW) and *C. autumnale* (16.84 mg/g DW). The total alkaloid content in the seeds was measured as 2.70 mg/g DW for *C. autumnale*, 1.16 mg/g DW for *C. diampolis*, and 1.32 mg/g DW for *C. bivonae*. Demecolcine concentrations varied among the species, with 0.10 mg/g DW in *C. autumnale*, 0.16 mg/g DW in *C. diampolis*, and 0.26 mg/g DW in *C. bivonae*. Colchicine levels were highest in *C. autumnale* seeds (2.70 mg/g DW), supporting its role in deterring herbivores during seed development, as noted by Yagi, et al. [32]. Unsaturated fatty acids, such as oleic acid (4.78 mg/g DW) and linoleic acid (5.35 mg/g DW) in *C. autumnale*, were identified as vital components of the lipid bilayer, essential for maintaining cellular membrane fluidity and adaptability. These findings are consistent with Asmaey, et al. [33], who identified these fatty acids as prominent in the methanolic extracts of *C. palaestinum*. Similarly, Hailu, et al. [34] detected saturated and unsaturated fatty acids in *C. autumnale* using GC-MS.

Leaves, as primary photosynthetic organs, contained high concentrations of metabolites associated with energy regulation and herbivore deterrence. In *C. autumnale*, significant metabolites included glyceric acid (4.41 mg/g DW), arabinose (3.25 mg/g DW), and galactose (18.52 mg/g DW), which play key roles in photosynthesis and energy storage. Additionally, oxalic acid was detected in *C. bivonae* leaves (0.47 mg/g DW), potentially involved in calcium regulation and herbivore deterrence. The total phenolic acid content in the leaves of *C. autumnale*, *C. diampolis*, and *C. bivonae* was 2.56 mg/g DW, 0.28 mg/g DW, and 3.86 mg/g DW respectively. Rocchetti, et al. [30] reported an average total phenolic acid content of 2.56 mg/g DW in *C. szovitsii*, while Senizza, et al. [31] described a total phenolic acid content of 0.28 mg/g DW in the leaves of *C. triphyllum*. The levels of total amino acids varied across species, with *C. bivonae* exhibiting the highest concentration (3.86 mg/g DW), followed by *C. diampolis* (2.76 mg/g DW) and *C. autumnale* (2.14 mg/g DW). These results underscore the dual role of leaves in energy capture and chemical defense.

Flowers of the three species demonstrated a unique metabolite profile, dominated by phenolic acids. The total phenolic acid content was 2.78 mg/g DW for *C. autumnale*, 3.14 mg/g DW for *C. diampolis,* and 2.94 mg/g DW for *C. bivonae*. These levels are consistent with those reported by Rocchetti, et al. [30] for *C. szovitsii* (2.28–3.27 mg/g DW) and Senizza, et al. [31] for *C. triphyllum* (0.16 mg/g DW). Organic acids, including glyceric, malic, and citric acid, were also detected significantly. The total alkaloid content in the flowers was 1.39 mg/g DW for *C. autumnale*, 0.53 mg/g DW for *C. diampolis*, and 0.44 mg/g DW for *C. bivonae*. Key fatty acids, such as palmitic acid and linoleic acid, were identified, consistent with findings by Baltacı, et al. [35], who noted their prominence in the flowers and stems of *C. autumnale*.

The fruit capsules displayed a distinctive metabolite profile, with the highest total phenolic acid content recorded in *C. diampolis* (4.54 mg/g DW), followed by *C. bivonae* (4.15 mg/g DW) and *C. autumnale* (3.49 mg/g DW). Organic acids were detected in notable quantities, with *C. bivonae* (11.63 mg/g DW DW) exhibiting the highest levels, followed by *C. diampolis* (11.07 mg/g DW) and *C. autumnale* (6.03 mg/g DW). Conversely, alkaloid concentrations in fruit capsules were consistently low across all three species.

Multivariate statistical analyses provided deeper insights into the unique metabolic profiles of different plant organs, highlighting their functional specialization. Techniques like Partial Least Squares Discriminant Analysis (PLS-DA) were crucial in identifying key metabolites, such as colchicine, demecolcine, and sucrose, which were principal factors in distinguishing the metabolic signatures of each organ. This differentiation underscores a significant degree of biochemical specialization tailored to the specific physiological roles of the organs. For instance, the clustering of corms and seeds metabolite profiles reflects their shared functions in energy storage and chemical defense. Corms act as reservoirs, accumulating essential compounds for survival under adverse conditions, while seeds leverage similar metabolites to maintain viability and deter herbivores during germination. The high levels of primary metabolites like sucrose and malic acid function as precursors or energy sources for alkaloid biosynthesis, as demonstrated in studies of other alkaloid-producing systems [26]. In contrast, clustering leaves, flowers, and fruit capsules emphasize their roles in energy capture, reproduction, and pollinator attraction. Leaves are metabolically optimized for photosynthesis and energy regulation, whereas flowers and fruit capsules are enriched with compounds that protect reproductive tissues and support seed dispersal.

This organ-specific metabolic differentiation highlights the plant’s ability to allocate resources strategically, optimizing each organ for its ecological and physiological roles. The findings not only reveal a high level of biochemical compartmentalization but also provide a framework for understanding how metabolic specialization supports the survival and adaptation of the species in diverse environments.

Comparative metabolite profiling of the three *Colchicum* species (*C. autumnale*, *C. bivonae*, and *C. diampolis*) underscores their distinct adaptations to specific ecological niches. For example, the corms of *C. diampolis* demonstrated elevated concentrations of 2-demethylcolchicine and gentisic acid, indicating a greater dependence on toxic alkaloids for defense compared to the other species. Similarly, the seeds of *C. bivonae* were rich in glucitol and demecolcine, which highlights their adaptation to xerothermic environments where robust chemical defenses and efficient water retention are vital for survival. These species-specific metabolic traits reflect their strategies to thrive in different ecological conditions.

### 3.3. Acetylcholinesterase Inhibitory Activities of Alkaloid Extracts from C. autumnale, C. bivonae, and C. diampolis Corms

According to numerous studies the strongest AChE inhibitors possess nitrogen in their molecules and are generally alkaloids and some terpenoids [29]. In the search for new natural AChE inhibitors, alkaloid extracts from *Colchicum* corms were evaluated for their anti-acetylcholinesterase activity. Colchicine, the primary alkaloid found in the corms of *C. autumnale* and *C. bivonae* (Table 2), showed no inhibitory activity against the AChE enzyme. Low positive correlations of acetylcholinesterase inhibitory activities with concentrations of other alkaloids found in the corm extracts (corigenine, autumnaline, and demecolcine), (Figure 13) demonstrated that alkaloids from *Colchicum* species cannot be considered as potential acetylcholinesterase inhibitors.

The AChE inhibitory activity of plants belonging to the *Colchicum* genus has not been extensively studied, with only limited reports available for species from Turkey [12] and Pakistan [36]. Consistent with our findings, Sevim et al. [12] reported that extracts from the seeds and corms of 104 *Colchicum* samples of Turkish origin showed no significant activity against the acetylcholinesterase enzyme. Among these, only five extracts demonstrated AChE inhibitory activity, with the highest activity recorded in *C. variegatum* L. seeds (35.50 ± 2.26%). The authors attributed this inhibitory activity to non-colchicine alkaloids, such as demecolcine. Ahmad, et al. [36] reported that the crude methanolic extract of *Colchicum luteum* Baker exhibited low (29.30%) inhibition, while moderate activity was displayed by the n-butanol fraction (45.10%) and aqueous fraction (47.30%). However, stronger inhibitory activity was found in the chloroform (61.20%) and ethyl acetate (56.10%) fractions. These results were significantly higher than ours, though no detailed information was provided on the chemical constituents of the crude extract or the various fractions analyzed by Ahmad, et al. [36]. It is possible that these samples contained compounds other than alkaloids contributing to their observed high AChE inhibitory activity.

## 4. Materials and Methods

### 4.1. Plant Material

The plant materials of *C. autumnale* L., *C. bivonae* Guss., and *C. diampolis* Delip. et Česchm., namely flowers, leaves, corms, fruit capsules, and seeds, were collected from three natural populations (Table 2). The samples were collected under an official permit № 965/27.01.2023 from the Ministry of Environment and Water of Bulgaria. The samples were identified by Dr. Ivanka Semerdjieva (Agricultural University, Plovdiv, Bulgaria/Institute of Biodiversity and Ecosystem Research at the Bulgarian Academy of Sciences). Voucher specimens of target species were deposited at the herbarium at Agricultural University (SOA), Plovdiv, Bulgaria. The herbarium specimen numbers were as follows: *C. bivonae*—063585; *C. autumnale*—063586-063588; *C. diampolis*—063589-063590.

Approximately twenty plants were sampled from the same population. The collected plant materials were thoroughly cleaned by rinsing with tap water and then with distilled water to remove soil and other contaminants. The plant parts, including flowers, leaves, fruit capsules, seeds, and corms were carefully separated and then lyophilized in a laboratory freeze dryer Alpha 1-2 LSCbasic, Martin Christ GmbH, Osterode am Harz, Germany. The dried plant materials were ground into a fine powder using a sample disruption/homogenizer system QIAGEN TissueLyser II, Retsch GmbH, Haan, Germany. Powdered materials were used for extract preparation.

### 4.2. Histochemical Analyses

Transverse sections and semi-permanent preparations were made using corms, flowers, and seeds of *C. autumnale*, *C. bivonae,* and *C. diampolis*. Subsequently, the sections of the studied plant organs were stained with Dragendorff’s reagent as described by Haist, et al. [25]. According to this method, the alkaloid precipitant reagent was applied at a rate of one drop per section, for 5 min, followed by three successive washes with distilled water [25]. Unstained (control) and colored sections were mounted in 50% glycerin. The microscopic slides were observed using the light microscope Motic DMA (Xiamen, China) and a microscope equipped with a Moticam A5, 5MP live resolution (flowers), and Stereo Microscope Motic DM 143 (Xiamen, China) (corms, seeds) in the laboratory of the Department of Botany and Agrometeorology at Agricultural University, Plovdiv, Bulgaria.

### 4.3. Extraction Methods

In this study, different extraction techniques employing various solvents were evaluated.

#### 4.3.1. Polar Metabolites and Lipids

The extraction procedure for the polar and lipid fractions was carried out following the methods outlined by Lisec, et al. [37], Roessner, et al. [38], and our previous study [39] with some modifications to optimize the process. Briefly, 50.0 mg of lyophilized material from each sample was mixed with 500.0 µL of methanol, 50.0 µL of ribitol, 50.0 µL of 2,5-dichloro-4-hydroxybenzoic acid, and 50.0 µL of n-nonadecanoic acid (internal standards, 1 mg/mL each in methanol for polar and non-polar metabolite quantification, respectively). The solution was heated on a Thermo Shaker TS-100 (Analytik Jena AG, Jena, Germany) at 70 °C and 300 rpm for 30 min. After cooling to room temperature, 200.0 µL of water and 500.0 µL of chloroform were added. The mixture was centrifuged at 13,000 rpm for 5 min at 22 °C (Beckman Coulter, Indianapolis, IN, USA). Three separate fractions were collected and vacuum-dried at 40 °C using a centrifugal vacuum concentrator (Labconco Centrivap, Kansas City, MO, USA): 300.0 µL of the upper phase for the analysis of amino acids, organic acids, and sugars (A); 300.0 µL of the upper phase for phenolic acids (B); and 300.0 µL of the lower phase for fatty acids (C).

Before GC-MS analysis, the three fractions underwent derivatization. For fraction A, the dried residue was treated with 300.0 µL of a methoxyamine hydrochloride solution (20 mg/mL in pyridine), followed by heating on a Thermo Shaker at 90 °C and 300 rpm for 1 h. After that, 100.0 µL of N, O-Bis(trimethylsilyl)trifluoroacetamide (BSTFA) was added, and the mixture was heated again at 75 °C and 300 rpm for 1 h. Finally, 1.0 µL of the derivatized solution was injected into the GC-MS. For fraction B, the dried residue was mixed with 1.0 mL of 1 M NaOH and left overnight at room temperature in a dark place. After adjusting the pH to 2 with 1 M HCl, the mixture was heated at 96 °C and 300 rpm for 1 h. Once cooled, the mixture was extracted three times with 500.0 mL of diethyl acetate, and the combined organic layers were vacuum-dried at 40 °C using a centrifugal vacuum concentrator. Pyridine (100.0 µL) and BSTFA (100.0 µL) were then added, and the mixture was heated at 75 °C and 300 rpm for 40 min. Finally, 1.0 µL of the derivatized solution was injected into the GC-MS. For fraction C, 1.0 mL of 2% H_2_SO_4_ in methanol was added to the dried residue and heated at 96 °C and 300 rpm for 1 h. After cooling, the mixture was extracted three times with 10.0 mL of n-hexane, and the combined organic layers were vacuum-dried at 40 °C. After drying, 100.0 µL of pyridine and 100.0 µL of BSTFA were added, and the mixture was heated at 75 °C and 300 rpm for 40 min before being injected into the GC-MS.

Standard substances for peak identification were dissolved in methanol at a concentration of 10 mg/mL. A 5 μL aliquot of the standard solution was evaporated under vacuum and derivatized with 50 μL of 20 mg/mL methoxyamine hydrochloride in pyridine and 50 μL of BSTFA, according to the procedure described above. The same procedures were applied for the peak identification of fatty acid methyl esters (ME), and phenolic acids trimethylsilyl esters (TMS), with the appropriate derivatization method. Derivatization was not performed for alkaloids. To assess the efficiency of the extraction procedure, recovery rates of various standard metabolites were determined by adding authentic metabolite standards to the tissue sample at the start of the extraction. The standards were added in a threefold excess compared to the measured endogenous concentrations. Recovery estimates were as follows: 119% for isoleucine, 97% for leucine, 102% for valine, 107% for malic acid, 113% for fructose, 98% for glucose, 101% for sucrose, and 105% for glucitol, 110% for benzoic acid, 107% for malic acid, 99% for palmitic acid, 104% for linoleic acid, and 109% for colchicine, respectively.

#### 4.3.2. Alkaloids

To extract alkaloid fractions from lyophilized plant materials, the following procedure outlined by Bharathi, et al. [40] was adopted. Initially, 50 mg of plant material was combined with 50.0 µL of galantamine, used as an internal standard (IS). The mixture was extracted with 1.0 mL of petroleum ether, with frequent shaking for 1 h, to remove non-polar compounds. After centrifugation, the supernatant was discarded, and the remaining solid residue was air-dried. Subsequently, the residue was subjected to a second extraction with 1.0 mL of dichloromethane at room temperature for 30 min, with periodic shaking to ensure thorough mixing. Afterward, 50.0 µL of a 25% ammonia solution was introduced to the extract, and the pH was adjusted to 8. The mixture was vigorously agitated for 10 min to ensure proper mixing and then left undisturbed for 30 min to facilitate the release of the target compounds. The mixture was then filtered to remove solids, and the filtrate was centrifuged to clarify the solution. The solvent from the centrifuged extract was evaporated to dryness under suitable conditions. Finally, the resulting dry residue was reconstituted in 100.0 µL of chloroform, producing a concentrated sample ready for GC-MS analysis without requiring derivatization.

### 4.4. GC-MS Profiling of Polar Metabolites, Lipids, and Alkaloids

The untargeted phytochemical profile of the different Colchicum species extracts was investigated through GC-MS.

The analysis was performed using a 7890A gas chromatograph (Agilent Technologies, Santa Clara, CA, USA) coupled with a 5975C mass-selective detector (Agilent Technologies, Santa Clara, CA, USA). Separation was achieved on a 30 m × 0.25 mm (i.d.) DB-5ms silica-fused capillary column with a 0.25 µm poly(dimethylsiloxane) coating as the stationary phase. Helium was used as the carrier gas at a flow rate of 1.0 mL/min. The injector and transfer line temperatures were maintained at 250 °C. The oven temperature for polar metabolites and alkaloids was programmed to start at 100 °C for 2 min, increase at a rate of 15 °C/min to 180 °C, then increase at a rate of 5 °C/min to 300 °C and finally hold for 10 min. In the case of lipids: initially 60 °C for 2 min then 5 °C/min to 300 °C for 1 min for 10 min. The injection volume was 1 µL, with injections carried out in split mode (10:1). The mass spectrometer operated in electron impact (EI) mode at 70 eV, scanning a mass range of 50–550 *m*/*z*.

To calculate retention indices (RI), a mixture of aliphatic hydrocarbons (C_10_–C_40_) from Sigma was injected under the same temperature program. AMDIS software (Automated Mass Spectral Deconvolution and Identification System, NIST, Gaithersburg, MD, USA), version 2.73 utilized a standard n-hydrocarbon calibration mixture to determine RIs. Compound identification was accomplished by comparing the RIs and spectral data with references from a custom reference library, the Golm Metabolome Database (http://gmd.mpimp-golm.mpg.de/analysisinput.aspx accessed 6 June 2024) [41] and the NIST’08 database (National Institute of Standards and Technology, Gaithersburg, MD, USA) [42].

### 4.5. Analysis of Acetylcholinesterase Inhibitory Activity

Acetylcholinesterase (AChE) inhibitory method was performed by using a colorimetric method described by López, et al. [43] with slight modifications, described previously by Ivanov, et al. [44]. 0.86 U AChE (type VI-S; Sigma-Aldrich, St. Louis, MO, USA) was dissolved in a volume of 1.0 mL 50 mmol phosphate buffer (pH 8.0), supplied with 0.15 mol NaCl and 0.05% (*v*/*v*) Tween 80 (Duchefa Biochemie, Haarlem, The Netherlands). The prepared enzyme solution (20 μL) was added into 2.0 mL 50 mmol phosphate buffer (pH 8.0) and mixed with 20 μL of analyzed alkaloid extracts. The samples were incubated for 20 min at 4 °C in darkness, then the reaction was started by adding 20 μL 6.0 mmol (in 50 mmol phosphate buffer with pH 7.0) acetylthiocholine iodide (Sigma) and 20 μL 5.0 mmol (50 mmol phosphate buffer with pH 7.0) 5,5′-dithiobis-(2-nitrobenzoic acid) (DTNB, Sigma). Samples were vortexed and incubated at 37 °C for 20 min in darkness. After the reaction time, the samples were cooled down in ice and 20 μL of 1.8 mmol (50 mmol phosphate buffer pH 7.0). Eserine salicylate (Sigma) was added to inactivate the enzyme. A blank sample with pure methanol instead of essential oil was prepared, as well. Positive control samples were developed for blank and experimental samples, following the same procedure but the enzyme was fully inhibited by adding 20 μL of 1.8 mmol eserine salicylate solution before starting the enzyme reaction. Changes in the absorption of samples against their positive controls were measured at 405 nm wavelength.

### 4.6. Statistical Processing

All analyses were performed on three biological samples (n = 3) with three technical repeats each. The web-based platform Metaboanalyst 6.0 (https://new.metaboanalyst.ca/, accessed on 1 October 2024) [45] was used to analyze the generated data. The results are presented as mean values with standard deviations (±SD). One-way ANOVA combined with the Tukey post hoc test was used to analyze the data. The significant differences were accepted at *p* ≤ 0.05. Clustering analyses were performed using the Ward method in combination with Euclidean distances.

## 5. Conclusions

Comprehensive GC-MS metabolite profiling of *C. autumnale*, *C. bivonae*, and *C. diampolis* revealed significant differences in chemical composition specific to each species and organ, allowing clear differentiation between the species and facilitating their unique metabolic features. Histochemical analysis identified alkaloids concentrated in specifying tissues such as the endosperm of seeds, the parenchyma of corms, and the vascular bundles of flowers. The consistent presence of colchicine and its less toxic derivatives, such as demecolcine and 2-demethylcolchicine, phenolic compounds, and other bioactive metabolites underscored their significant pharmacological potential, particularly for applications in treating inflammatory, antimicrobial, and antitumor conditions. *C. bivonae* showed moderate acetylcholinesterase (AChE) inhibitory activity, while *C. diampolis* exhibited unique metabolic traits.

Our findings offer a robust foundation for biotechnological conservation of these valuable endemic species and their sustainable utilization. Advanced bioprocess engineering approaches, including bioreactor cultivation of corm-derived cell and tissue cultures, can facilitate the production of key tropolone alkaloids with low cytotoxicity and significant pharmaceutical potential. These efforts will support the large-scale production of bioactive compounds ensuring the preservation of endemic *Colchicum* species as invaluable genetic and pharmacological resources.

## Figures and Tables

**Table 1:** Acetylcholinesterase inhibitory activities of alkaloid extract from *Colchicum autumnale, Colchicum bivonae,* and *Colchicum diampolis* corms. The means that do not share a letter in superscript are significantly different (Tukey’s test, *p* < 0.01).

Sample	Organ	% Inhibition (Means ± SD, n = 3)
*Colchicum autumnale*	corms	14.82 ^b^ ± 0.64
*Colchicum bivonae*	corms	16.44 ^a^ ± 0.39
*Colchicum diampolis*	corms	11.82 ^c^ ± 0.39
Colchicine standard	-	0.00

**Table 2:** Plant material of *C. autumnale*, *C. bivonae*, and *C. diampolis* collected from three natural habitats in Bulgaria.

Species	Location	GPS Coordinates	Date/Organs
*C. autumnale*	Ribaritsa, Teteven Municipality	42°52′30.6″ N 24°20′01.9″ E	11 June 2023 (leaves, fruit capsules, and seeds) 11 October 2023 (flowers and corms)
*C. bivonae*	Slivnitsa, Kresna Municipality	41°41′21.6″ N 23°09′55.0″ E	29 May 2023 (leaves, fruit capsules, and seeds) 23 September 2023 (flowers and corms)
*C. diampolis*	Iskra, Karnobat Municipality	42°39′20.1″ N 26°54′02.9″ E	15 February 2023 (leaves, fruit capsules, and seeds) (flowers and corms) 13 June 2023

## Data Availability

Data are contained within the article and Supplementary Materials.

## Supplementary Materials

The following supporting information can be downloaded at: https://www.mdpi.com/article/10.3390/plants14020270/s1, Table S1: Metabolites identified in extracts from *Colchicum autumnale*, *Colchicum bivonae* and *Colchicum diampolis* plant organs.
